# Genetic and epigenetic silencing of mircoRNA-506-3p enhances COTL1 oncogene expression to foster non-small lung cancer progression

**DOI:** 10.18632/oncotarget.13501

**Published:** 2016-11-22

**Authors:** Shanqi Guo, Peiying Yang, Xingkang Jiang, Xiaojiang Li, Yuanyuan Wang, Xin Zhang, Binxu Sun, Yao Zhang, Yingjie Jia

**Affiliations:** ^1^ Department of Oncology, First Teaching Hospital of Tianjin University of Traditional Chinese Medicine, Tianjin, China; ^2^ Department of Urology, The Second Hospital of Tianjin Medical University, Tianjin, China

**Keywords:** non-small cell lung cancer, miR-506-3p, COTL1, UCA1, LncRNA

## Abstract

Although previous studies suggested that microRNA-506-3p (miR-506-3p) was frequently downregulated, and functioned as a tumor suppressor in several cancers, the biological role and intrinsic regulatory mechanisms of miR-506-3p in non-small cell lung cancer (NSCLC) remain elusive. The present study found miR-506-3p expression was downregulated in advanced NSCLC tissues and cell lines. The expression of miR-506-3p in NSCLC was inversely correlated with larger tumor size, advanced TNM stage and lymph node metastasis. In addition, we also found patients with lower expression of miR-506-3p had a poor prognosis than those patients with higher expression of miR-506-3p. Function studies demonstrated that aberrant miR-506-3p expression modulates tumor cell growth, cell mobility, cell migration and invasion *in vitro* and *in vivo*. Mechanistic investigations manifested that coactosin-like protein 1 (COTL1) was a direct downstream target of miR-506-3p. Knockdown of COTL1 mimicked the tumor-suppressive effects of miR-506-3p overexpression in A549 cells, whereas COTL1 overexpression enhanced the tumorigenic function in HCC827 cells. Importantly, we also found GATA3 transcriptionally actives miR-506-3p expression, and the long non-coding RNA urothelial carcinoma-associated 1 (UCA1) exerts oncogenic function in NSCLC by competitively ‘sponging’ miRNA-506. Together, our combined results elucidated genetic and epigenetic silencing of miR-506-3p enhances COTL1 oncogene expression to foster NSCLC progression.

## INTRODUCTION

Lung cancer is the leading cause of cancer mortality worldwide, with non-small cell lung cancer (NSCLC) constituting more than 85% [1]. Besides, patients with NSCLC were usually diagnosed at advanced stages [2]. Despite recent advance in diagnosis and treatment, the prognosis of patients with advanced disease remains unsatisfied until now [3]. Therefore, it is necessary to elucidate the molecular mechanisms which mediate the initiation and progression of NSCLC for exploring the novel diagnosis biomarkers and the new effective therapeutic strategies.

MicroRNA (miRNA) are an abundant class of endogenous, 18-25 nucleotide long, single-stranded noncoding RNA molecules, which inducing mRNA degradation or the inhibition of mRNA translation by binding to 3′ untranslated regions of target mRNAs [4]. Accumulating studies demonstrated that miR-506 was remarkably downregulated in several types of human cancer, including breast cancer, liver cancer, gastric cancer and colon cancer [5–8]. In lung cancer, however, it has been noted that there are still inconsistent and even conflicting results among the different reports. For example, Zhao et al. revealed that miR-506 acts as an anti-oncogenic miRNA in malignant transformation of human bronchial epithelial cells (16HBE-T) [9]. In contrast, Yin and colleagues reported that miR-506 upregulation occurs in 83% of NSCLC patients [10]. These different arguments potentially add to the current uncertainty of whether the aberrant expression of miR-506 in NSCLC is associated with malignancy or progression, and the underlying molecular mechanisms through which miR-506 exerts its oncogenic activity needs to be illustrated. Other than these, the upstream signaling responsible for miR-506's genetic and epigenetic regulation are still limited.

In the current study, we thus intended to elucidate the molecular mechanisms underlying the miR-506-3p in NSCLC progression. Overall, we demonstrated that downregulation of miR-506-3p in human NSCLC is strong correlated with aggressive clinical-pathological features and poor prognosis, and aberrant expression of miR-506-3p modulates the cell growth, cell mobility, cell migration and invasion as well as cell apoptosis *in vitro* and *in vivo*. Mechanically, we uncovered that miR-506-3p inhibits tumor progression by repressing coactosin-like protein 1 (COTL1) expression. Importantly, we also found that GATA3 transcriptional actives miR-506-3p expression, and the long non-coding RNA (lncRNA) urothelial carcinoma-associated 1 (UCA1) exerts oncogenic function in NSCLC by competitively ‘sponging’ miRNA-506. Therefore, this study pointed out that targeting miR-506-3p or its signaling molecules may provide a promising means of treating NSCLC.

## RESULTS

### Downregulated expression of miR-506-3p predicts poor prognosis in NSCLC patients

**Figure 1:**
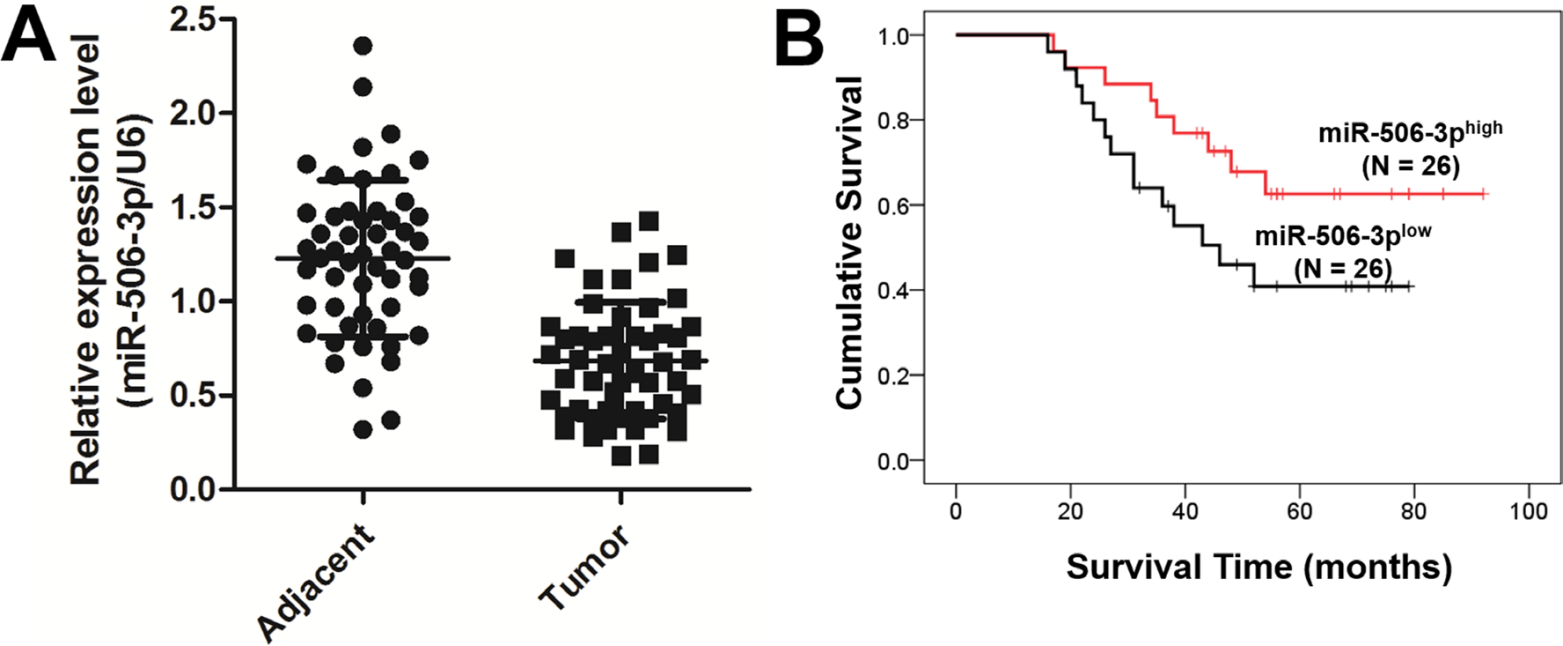
Downregulated expression of miR-506-3p predicts poor prognosis in NSCLC patients (**A**) Expression of miR-506-3p in 52 matched pairs of primary NSCLC tissues and their corresponding adjacent samples. The expression level of miR-506-3p was detected using qPCR and normalized against an endogenous control (U6) mRNA. (**B**) Patients with a lower expression of miR-506-3p had a poor prognosis than the patients with high expression of miR-506-3p.

To determine the significance of miR-506-3p in NSCLC, we examined the expression of miR-506-3p in NSCLC tissue by qRT-PCR. As showed in Figure 1A, miR-506-3p expression was significantly downregulated in 52 NSCLC specimens compared with their corresponding adjacent non-tumorous tissues (*P* < 0.05). To further identify clinical significance of miR-506-3p in NSCLC, we divided those patients into two groups, according to their average expression level. The Chi-square method indicated that the expression level of miR-506-3p was positively correlated with larger tumor size, advanced tumor-node-metastasis (TNM) stage and lymph node metastasis (*P* < 0.05), suggesting miR-506-3p might be a potential biomarker for NSCLC (Table 1). However, no significant correlation was observed between the abnormal expression of miR-506-3p and patients' age, gender, and smoking habits (Table 1). In addition, we also evaluated the potential effect of miR-506-3p expression on the clinical outcome of patients with NSCLC. The Kaplan-Meier method suggested that patients with lower expression of miR-506-3p had a poor prognosis than those patients with higher expression of miR-506-3p (Figure 1B, *P* < 0.05). The data collectively indicated that downregulation of miR-506-3p is closely associated with poor survival of patient with NSCLC.

**Table 1:** Relationship between miR-506-3p and clinicopathologic variables

Feature	Total number (*n*)	miR-506 expression		*P* value
		High (*n*)	Low (*n*)	
Gender				
Male	30	16	14	0.78
Female	22	10	12	
Age				
≥ 55	35	19	16	0.56
< 55	17	7	10	
Smoking history				
Yes	23	12	11	1.00
No	29	14	15	
Tumor size				
≥ 5	29	18	11	**0.03**
< 5	28	7	16	
TNM stage				
I + II	24	17	8	**0.03**
III + IV	28	9	18	
Lymph node metastasis				
Yes	32	11	19	**0.04**
No	10	15	7	

### Abnormal expression of miR-506-3p alters the growth of NSCLC cells

**Figure 2:**
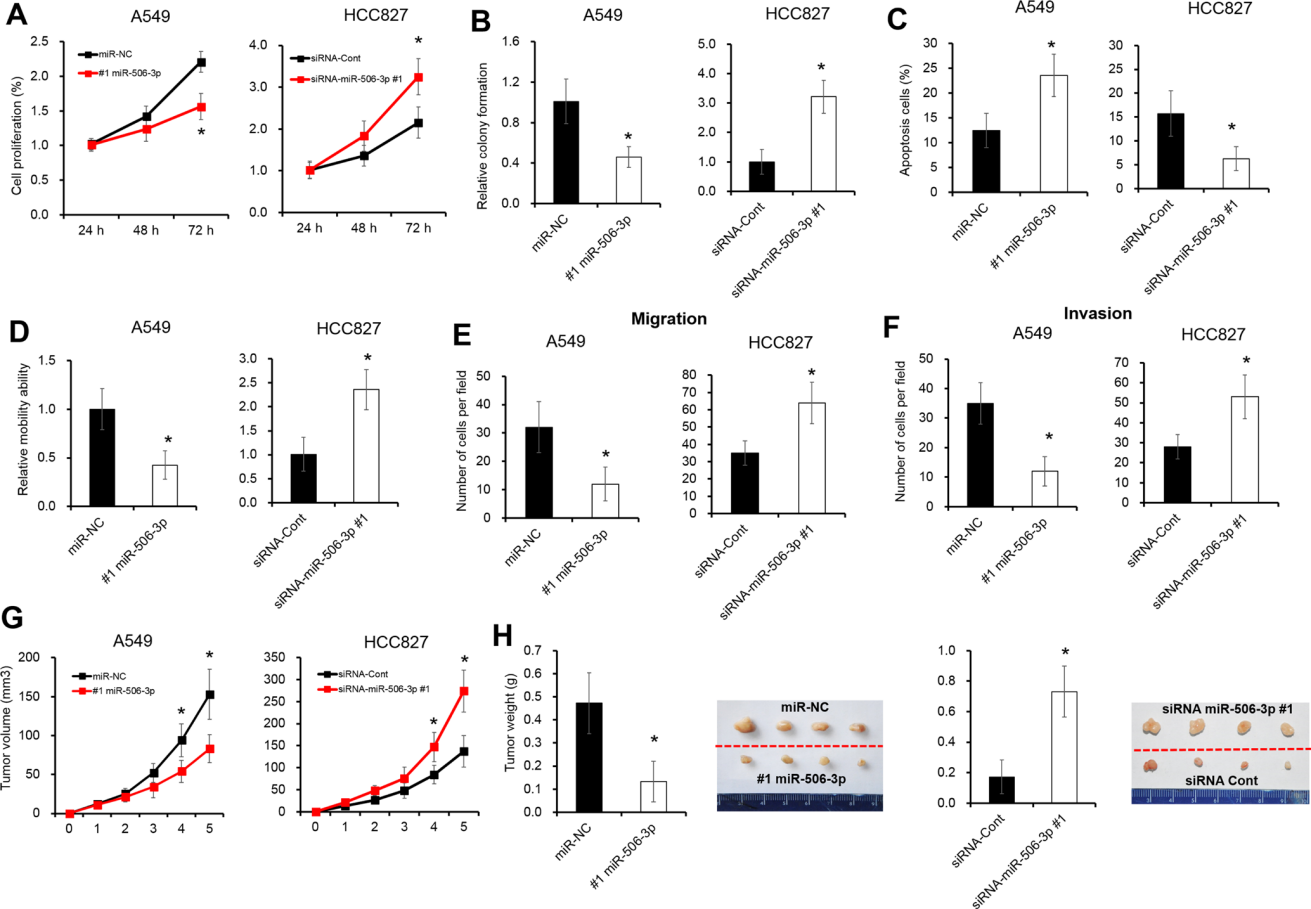
Abnormal expression of miR-506-3p alters the growth of NSCLC cells (**A**) Alarmar Blue assay showed that overexpression of miR-506-3p inhibits cell growth of A549 cells in 72 h, whereas inhibition of miR-506-3p in HCC827 cells promotes cell proliferation in 72 h. (**B**) Colony formation assay showed that colony ability of A549 cells was inhibited after treatment of miR-506-3p mimics in 6 days, while silencing of miR-506-3p in HCC827 cells promoted cell colony formation in 6 days. (**C**) FACS assay showed that overexpression of miR-506-3p promotes cell apoptosis of A549 cells in 48 h, whereas inhibition of miR-506-3p in HCC827 cells inhibits cell apotosis in 48 h. (**D**) Wound healing assay showed that cell mobility ability was inhibited when transfected by miR-506-3p mimics in A549 cells in 48 h, whereas silencing of miR-506-3p in HCC827 cells promotes cell mobility in 48 h. (**E**–**F**) Transwell assay showed that overexpression of miR-506-3p inhibits cell migration and invasion ability of A549 cells in 48 h, while inhibition of miR-506-3p in HCC827 cells promotes cell migration and invasion in 48 h. (**G**–**H**) The mean volume and weight of the xenograft tumors in miR-506-3p-A549 group was smaller than those of miR-NC-A549 group, whereas silencing of miR-506-3p in HCC827 cells promotes tumor weight than those of siRNA-miR-NC-HCC827 xenografted tumors. Asterisk (*) indicated *P* < 0.05.

We further assessed the expression level of miR-506-3p in NSCLC cell lines. As illustrated in Supplementary Figure S1A, the mRNA levels of miR-506-3p were significantly downregulated in three different NSCLC cell lines (e.g. A549, SPC-A1 and HCC827 cells), when compared with that of non-tumorigenic bronchial epithelium cell line BEAS-2B cells (*P* < 0.05). To explore the biological role of miR-506-3p in NSCLC, two NSCLC cell lines A549 and HCC827 cells were selected to establish cell lines with overexpression or knockdown of miR-506-3p (Supplementary Figure S1B–S1C). A cell proliferation and colony formation assays revealed that overexpression of miR-506-3p in A549 cells significantly decreased cell proliferation, whereas silencing expression of miR-506-3p greatly increased cell growth in HCC827 cells (Figure 2A–2B, *P* < 0.05). Next, we further evaluated the effect of miR506-3p on cell apoptosis using Annexin V-FITC and PI staining. Flow cytometry analysis showed that miR-506-3p overexpression significantly induced cell apoptosis in A549 cells, while downregulation of miR-506-3p in HCC827 cells decreased cell apoptosis (Figure 2C, *P* < 0.05). Moreover, we also explored the biological behavior of miR-506-3p in mobility, migration and invasion of NSCLC cells by wound-healing and transwell assay. Ectopic expression of miR-506-3p in A549 cells promoted the ability of cell mobility, invasion and migration, whereas silencing expression of miR-506-3p in HCC827 cells inhibited the ability to mobility, migration and invasion (Figure 2D–2F, *P* < 0.05). Consistent to *in vitro* study, we also found tumor growth was substantially inhibited by 50% in miR-506-3p transfected A539 cells, while downregulation of miR-506-3p in HCC827 cells promoted tumorigenicity by 2.3-fold in nude mice (Figure 2G–2H, *P* < 0.05). These results together showed that abnormal expression of miR-506-3p alters the growth of NSCLC cells.

### miR-506-3p directly targets to COTL1 3′UTR

**Figure 3:**
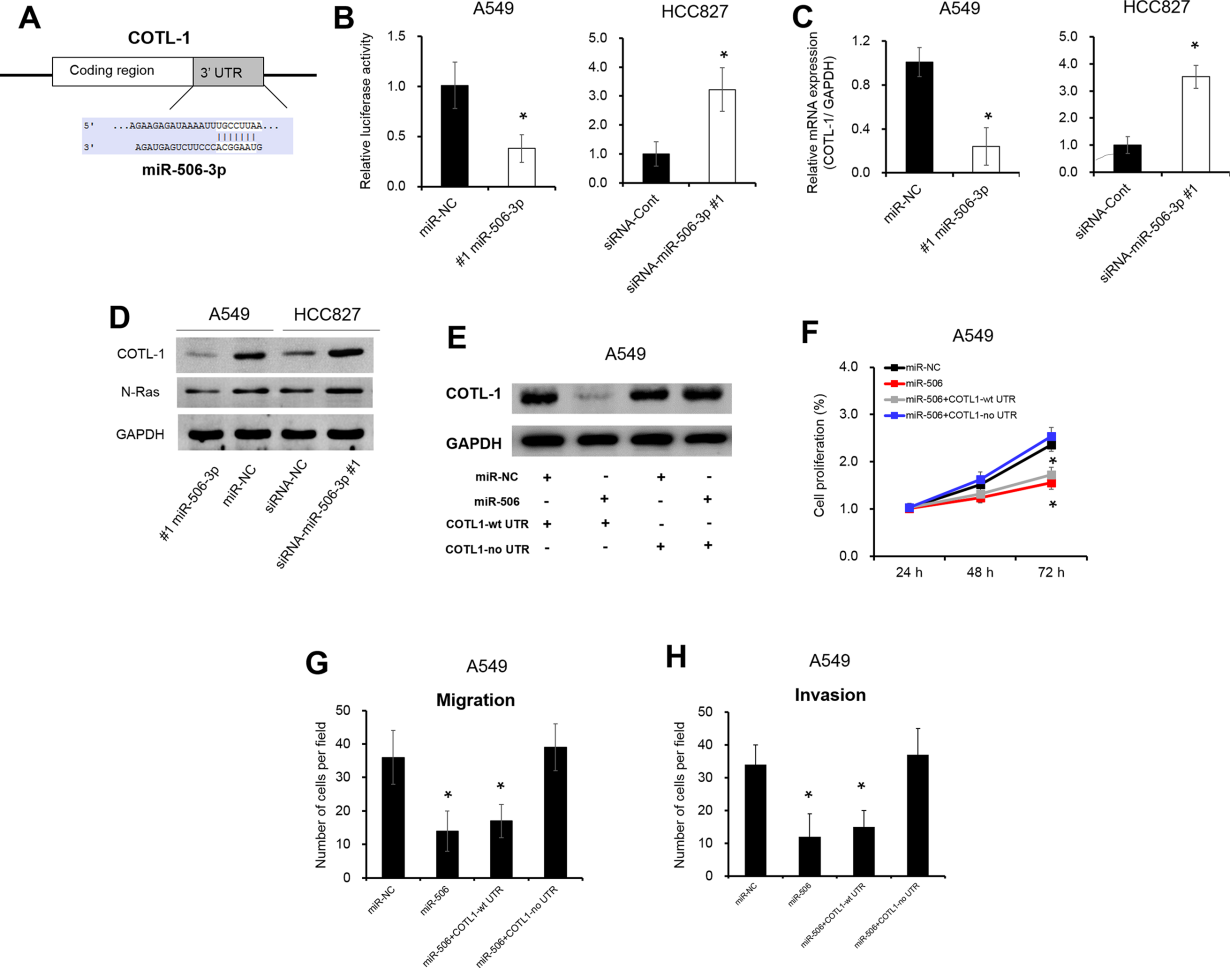
miR-506-3p directly targets to COTL1 3′UTR (**A**) Targetscan software indicated that COTL1 was a potential target of miR-506-3p. (**B**) Luciferase reporter assay showed that COTL1 was a direct downstream target of miR-506-3p in A549 and HCC827 cells. (**C**–**D**) Overexpression of miR-506-3p inhibits the mRNA and protein expression of COTL1 in A549 cells, whereas silencing of miR-506-3p in HCC827 cells promotes COTL1 mRNA and protein levels. The oncogene N-Ras, a direct gene of miR-506-3p, has been acted as a positive control. (**E**) Western blot analysis demonstrated that COTL1-no 3′UTR abrogates that miR-506-3p mediated induction of COTL1, but COTL1-wt 3′UTR was found to decrease COTL1 protein expression in A549 cells. (**F**) Alarmar Blue assay showed that COTL1-no 3′UTR promoted the proliferation of miR-506-3p transfected A549 cells; however, COTL1-wt 3′UTR cells had no significant impact in the miR-506-3p transfected A549 cells. (**G**–**H**) Transwell assays showed that COTL1-no 3′UTR in A549 cells increased migration and invasion in miR-506-3p transfected cells; however, COTL1-wt 3′UTR cells had no significant impact in the miR-506 transfected cells. Asterisk (*) indicated *P* < 0.05.

To investigate the underlying mechanisms by which miR-506-3p exerts its function, we searched for potential miR-506-3p targeting using an online software (TargetScan bioinformatics algorithm). COTL1 was identified as a potential target of miR-506-3p based on putative target sequences at position 669-676 of COTL1 3′UTR (Figure 3A). To verify post-transcriptional regulation of COTL1 by miR-506-3p, we cloned the COTL1 3′UTR fragment into the downstream of a pGL3-Basic vector. As showed in Figure 3B, overexpression of miR-506-3p in A549 cells repressed the luciferase activity by 50% when compared with their negative control (*P* < 0.05). On the contrary, silencing expression of miR-506-3p in HCC827 cells increased luciferase activity by 3.1-fold (Figure 3B, *P* < 0.05). To this end, we looked into possible direct regulation of COTL1 by miR-506-3p. As expected, ectopic expression of miR-506-3p in A549 cells reduced COTL1 expression in mRNA expression and protein levels, and downregulated miR-506-3p expression in HCC827 cells increased the mRNA and protein levels of COTL1 (Figure 3C–3D, *P* < 0.05). The oncogene N-Ras, a direct gene of miR-506-3p, has been acted as a positive control [9]. Ectopic expression of miR-506-3p in A549 cells decreased N-Ras protein level, while silencing of miR-506-3p in HCC827 promoted N-Ras expression (Figure 3D). Besides, we also transfected A549 cells with expression vectors encoding COTL1 with or without its 3′UTR (COTL1-wt 3′UTR and COTL1-no 3′UTR). Figure 3E showed that overexpression of COTL1-no 3′UTR abrogated the miR-506-3p-mediated suppression on COTL1 protein expression, while overexpression of COTL1-wt 3′UTR was found to decrease COTL1 protein level. Furthermore, overexpression of COTL1-no 3′UTR significantly increase the proliferation, migration and invasion in miR-506-3p transfected A549 cells, whereas overexpression of COTL1-wt 3′UTR had no significant impact on miR-506-3p transfected A549 cells (Figure 3F–3H, *P* < 0.05). These combined data thus verified a direct regulation of COTL1 by miR-506-3p binding to the 3′UTR of COTL1 mRNA.

### Aberrant expression of COTL1 governs NSCLC cell growth

**Figure 4:**
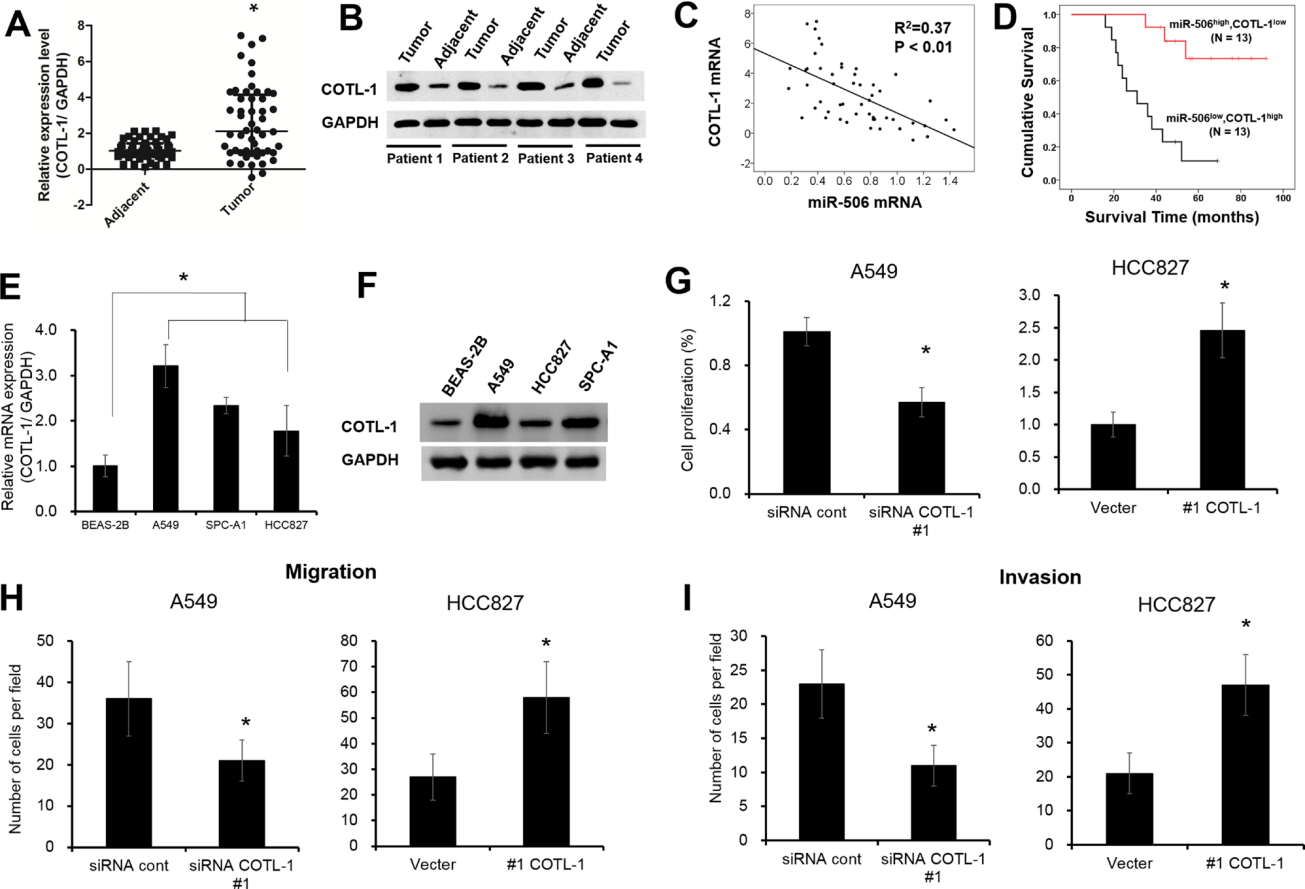
Aberrant expression of COTL1 governs NSCLC cell growth (**A**–**B**) COTL1 mRNA and protein levels were upregulated in NSLCL tissues compared with their corresponding adjacent samples. (**C**) miR-506 was inversely related to COTL1 mRNA in NSLCL tissues. (**D**) Kaplan-Meier survival curves depicted that patients with lower miR-506-3p and higher COTL1 expression had a poor prognosis than that with the patients with high miR-506-3p and lower COTL1 expression. (**E**–**F**) The mRNA and protein level of COTL1 were upregulated in A549, HCC827 and SPC-A1 cells compared with that of BEAS-2B cells. (**G**–**I**) COTL1 knockdown inhibited cell proliferation, migration and invasion ability in A549 cells, whereas COTL1 overexpression in HCC827 cells promotes cell growth, migration and invasion. Asterisk (*) indicated *P* < 0.05.

Next, we also found the mRNA and protein levels of COTL1 were significantly up-regulated in NSCLC tissues compared with adjacent samples (Figure 4A–4B, *P* < 0.05). Spearman's correlation analysis showed that miR-506-3p expression was negatively correlated with COTL1 mRNA expression in NSCLC tissues (Figure 4C, *P* < 0.05). Moreover, we also found that patients with lower miR-506-3p and higher COTL1 expression had a poor prognosis than those patients with higher miR-506-3p and lower COTL1 expression (Figure 4D, *P* < 0.05). In addition, the mRNA and protein levels of COTL1 were also greatly upregulated in three different NSCLC cell lines (e.g. A549, HCC827 and SPC-A1 cells), when compared with that of non-tumorigenic bronchial epithelium cell line BEAS-2B cells (Figure 4E–4F, *P* < 0.05). To further elucidate the contribution of COTL1 to cell growth, we assayed A549 cells after transduction with siRNA COTL1 (Supplementary Figure S2A). Compared with control, the cell growth was restrained by 40% in COTL1 knockdown cells (Figure 4G, *P* < 0.05). Similarly, COTL1 knockdown in A549 cells inhibited cell migration and invasion by 50% and 55%, respectively (Figure 4H–4I, *P* < 0.05). Meanwhile, we also restored the expression of COTL1 expression in HCC827 cells by transfecting COTL1 plasmids (Supplementary Figure S2B). Result showed that overexpression of COTL1 promoted cell proliferation, migration and invasion, which enhanced the tumorigenic function (Figure 4G–4I, *P* < 0.05). These results collectively demonstrated that aberrant expression of COTL1 governs NSCLC cell growth.

### Reduced GATA3 expression transcriptionally inhibits miR-506-3p level in NSCLC

**Figure 5:**
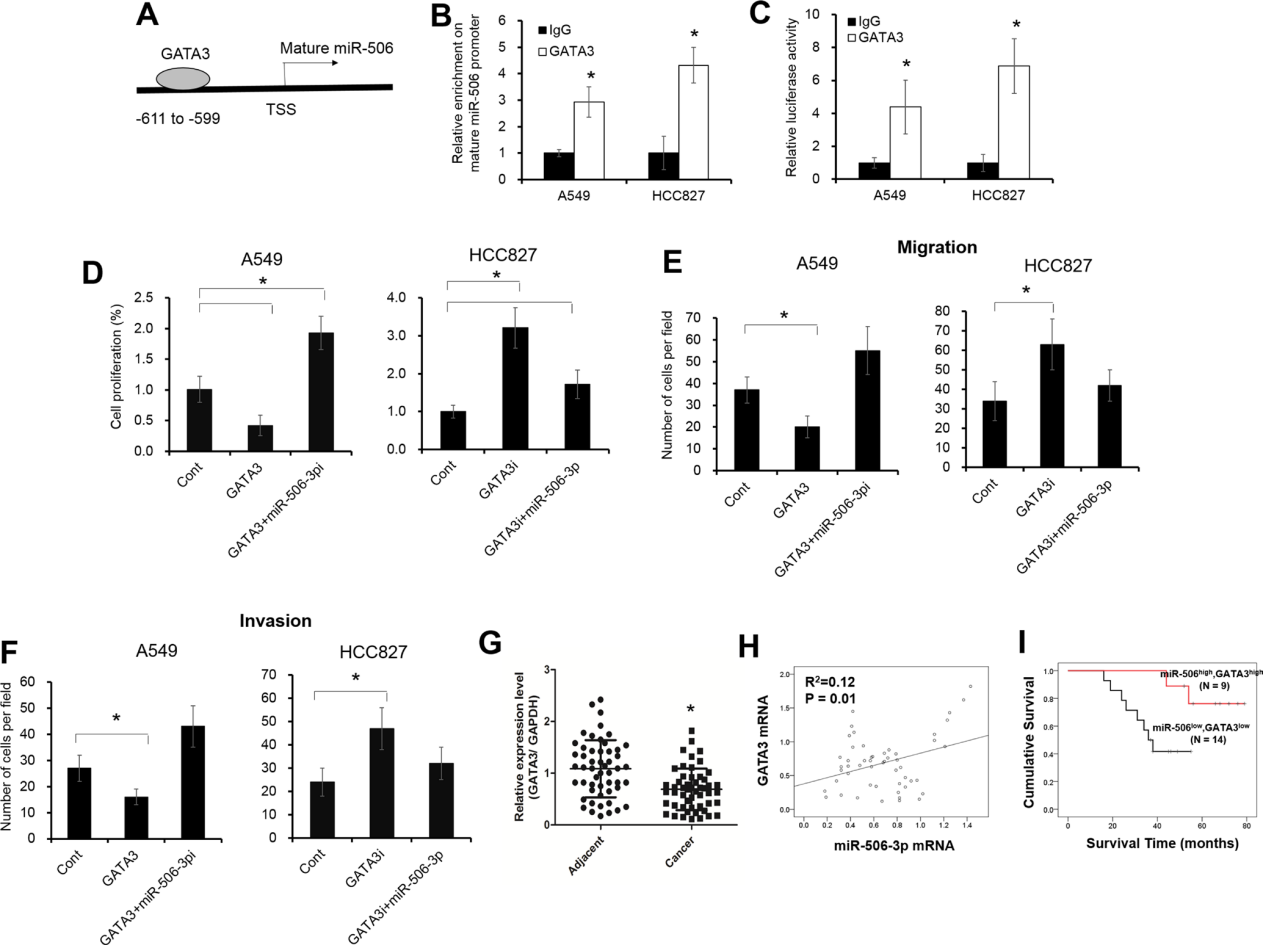
GATA3 transcriptionally modulates miR-506-3p expression in NSCLC (**A**) The putative GATA3 binding site in mature miR-506 promoter regions. (**B**) ChIP assay showed that GATA3 expression was increased in the promoter of mature miR-506 in A549 and HCC827 cells. (**C**) Luciferase activity of pGL3-miR-506-3p was increased in GATA3-transfected A549 cells and HCC827 cells. (**D**–**F**) Inhibition of miR-506-3p (miR-506i) attenuated the decreased cell proliferation, migration and invasion of GATA3 overexpression in A549 cells, whereas overexpression miR-506-3p inhibited the elevated cell growth, migration and invasion of GATA3 knockdown (GATA3i) in HCC827 cells. (**G**) The mRNA levels of GATA3 were decreased in NSCLC tissues compared with their adjacent samples. (**H**) Spearman correlation analysis revealed that GATA3 expression was positively correlated with miR-506-3p mRNA in NSCLC tissues. (**I**) Kaplan-Meier survival curves depicted that patients with lower miR-506-3p and lower GATA3 expression had a poor prognosis than that with the patients with higher miR-506-3p and higher GATA3 expression. Asterisk (*) indicates *P* < 0.05.

To look into how miR-506-3p expression was downregulated in NSCLC, we hypothesized that miR-506-3p expression might be regulated by several unknown transcriptional factors. Based on online bioinformatics analysis (Promo dataset), we found that the promoter of mature miR-506 contains a putative GATA3 binding sites from −599 to −611 (Figure 5A). We next endeavored to elucidate how GATA3 affect the transcriptional of the miR-506 promoter. The chromatin imunoprecipitation (ChIP) assay showed that significant enrichment was found within the promoter of mature miR-506 upon GATA3 antibody precipitation (Figure 5B, *P* < 0.05; Supplementary Figure S3A). Moreover, to further evaluate the contribution of GATA3 in miR-506-3p expression, we first transfected GATA3 siRNA or plasmids in NSCLC cells (Supplementary Figure S3B–S3C). The luciferase activity and mRNA levels of miR-506-3p was significantly elevated in GATA3-transfected A549 cells, whereas silencing expression of GATA3 in HCC827 cells decreased the luciferase activity and mRNA expression of miR-506-3p (Figure 5C, *P* < 0.05; Supplementary Figure S3D–3E). A number of miRNAs were reported as direct targets of GATA3, such as miR-29b, miR-503 [27, 28]. As previous described, elevated GATA3 expression promotes miR-29b and miR-503 levels in A549 cells, while inhibition of GATA3 in HCC827 cells decreases miR-29b and miR-503 expression (Supplementary Figure S3D–3E). Besides, we also found inhibition of miR-506-3p significantly attenuated the suppression of cell proliferation, migration and invasion in GATA3-transfected A549 cells (Figure 5D–5F, *P* < 0.05). On the contrary, ectopic expression of miR-506-3p decreased the cell growth, migration and invasion in HCC827 cells upon GATA3 knockdown (Figure 5D–5F, *P* < 0.05). Moreover, we also demonstrated that GATA3 was significantly downregulated in NSCLC samples, and was positively correlated with miR-506-3p levels (Figure 5G–5H, *P* < 0.05). Patients with lower GATA3 and lower miR-506-3p had a poor prognosis compared with those patients with higher GATA3 and higher miR-506-3p levels (Figure 5I, *P* < 0.05). These combined data demonstrated that GATA3 was transcriptionally actives miR-506-3p expression in NSCLC.

### Elevated UCA1 expression acts as a molecular sponge for miR-506-3p

**Figure 6:**
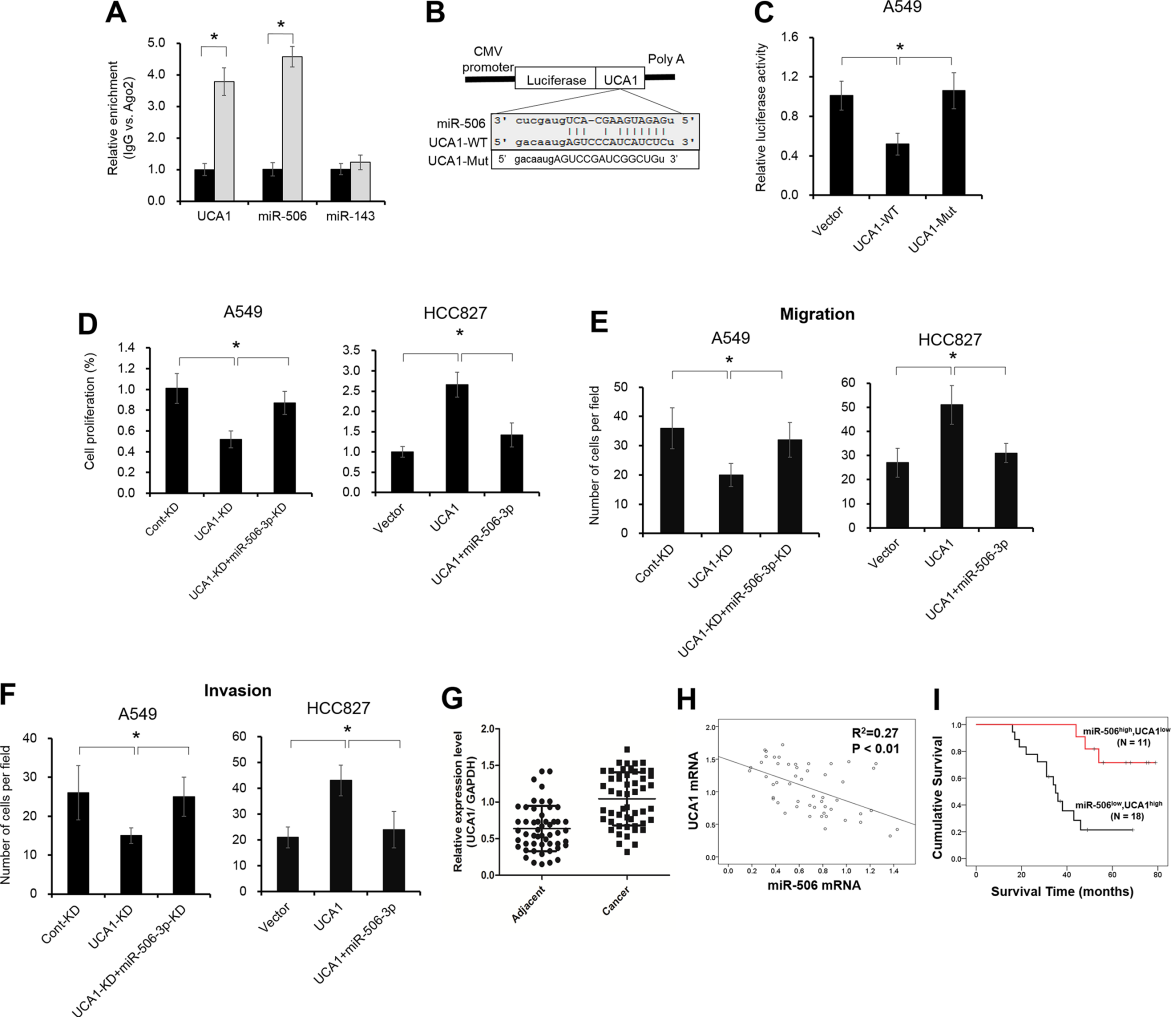
UCA1 acts as a molecular sponge for miR-506-3p (**A**) The association between UCA1, miRNA-506-3p and Ago2 was ascertained by analyzing A549 cells lysates by RNA immunoprecipitation with an Ago2 antibody. miR-143 act as a negative control. (**B**) Putative miR-506-3p binding sequence of UCA1 RNA. Mutation was generated on the UCA1 RNA sequence in the complementary site for the seed region of miR-506-3p. (**C**) A549 cells were transfected with the vector, wild-type UCA1 (UCA1-Wt) or mutant UCA1 (UCA1-Mut) with a mutation of the miRNA binding sites. Luciferase activity was measured using a dual-luciferase reporter gene assay system after 48 h transfection. (**D**–**F**) Inhibition of miR-506-3p (miR-506i) attenuated the decreased proliferation, migration and invasion effect of UCA1 knockdown (UCA1-KD) in A549 cells, whereas overexpression miR-506-3p inhibited the elevated cell growth, migration and invasion of UCA1-transfected HCC827 cells. (**G**) UCA1 mRNA levels were upregulated in NSLCL tissues compared with their corresponding adjacent samples. (**H**) miR-506-3p was inversely correlated with UCA1 mRNA in NSLCL tissues. (**I**) Kaplan-Meier survival curves depicted that patients with lower miR-506-3p and higher UCA1 expression had a poor prognosis than that with the patients with high miR-506-3p and lower UCA1 expression. Asterisk (*) indicated *P* < 0.05.

Previous studies indicated that lncRNA contributes to NSCLC progression by function as competing endogenous RNA (ceRNA) to specific miRNAs. To examine whether miR-506-3p makes a similar mechanism in NSCLC, we predicted miRNA target sites using online microRNA-target program (miRcode), and found out UCA1 with relevant binding sites in miR-506-3p. It is well reported that miRNA exerts its function by binding to Ago2. To confirm whether UCA1 interacts miR-506-3p in Ago2, we performed RNA immunoprecipitation (RIP) to detect the UCA1 and miR-506-3p in the Ago2 pellet. As illustrated in Figure 6A, miR-506-3p and UCA1 were significantly enriched almost 4-fold, indicating UCA1 may play a critical role in deregulation of miR-506-3p (*P* < 0.05). Besides, we further constructed pGL3 luciferase reporter gene containing UCA1, which contains wild-type or mutated-type miR-506-3p binding sites. Results showed that UCA1-mut construct no longer suppressed miR-506-3p expression, supporting that miR-506-3p is a UCA1 targeting miRNA (Figure 6B–6C, *P* < 0.05). To validate the importance of miR-506-3p binding in UCA1 promoting NSCLC progression, we also investigated the biological behavior of UCA1 in NSCLC cells by loss– and gain- experiments (Supplementary Figure S4A–4B). Elevated UCA1 expression in A549 cells promoted miR-506-3p expression, whereas decreased UCA1 expression in HCC827 cells inhibited miR-506-3p expression (Supplementary Figure S4C–4D). Besides, inhibition of miR-506-3p attenuated the decreased cell proliferation, migration and invasion effect of UCA1 knockdown in HCC827 cells, whereas ectopic expression of miR-506-3p increased cell growth, migration and invasion in A549 upon UCA1 overexpression (Figure 6D–6F, *P* < 0.05). These results indicated that UCA1 promotes tumor growth (at least in part) via competitively binding miR-506-3p. Moreover, we also found that the expression of UCA1 in NSCLC samples was significantly higher compared to that of adjacent tissues, and UCA1 level was negatively associated with miR-506-3p expression (Figure 6G–6H, *P* < 0.05). Patients with higher UCA1 and lower miR-506-3p had a poor prognosis than those patients with lower UCA1 and higher miR-506-3p (Figure 6H, *P* < 0.05). All together, these data indicated that elevated UCA1 exerts its oncogenic functions through competitively ‘sponging’ miRNA-506-3p.

## DISCUSSION

miRNAs have been reported to play an essential role in regulating of various cellular process, including cell growth, differentiation, apoptosis, and organ development. Emerging evidence indicated that numerous miRNA participated in the regulating of NSCLC initiation and progression [3, 4, 11]. For example, Liu and colleagues found miR-100 functions as a tumor suppressor by post-transcriptionally regulating PLK1 [12]. Moreover, Luo et al. reported that miR-449a inhibits cell proliferation, migration and invasion by targeting c-Met [13]. Zhang et al noted that miR-152 represses cell invasion by targeting neuropilin-1 [14]. Shan et al. found miR-153 restrains cell proliferation, migration and invasion of NSCLC cells through inhibiting ADAM19 expression [15]. Yang et al. demonstrated that miR-137 decreases cell migration and invasion by targeting BMP7 [16]. Although significant efforts have been made, the potential role of miRNA in NSCLC as well as underlying molecular mechanisms remained elusive, and should be closely scrutinized.

miR-506 belongs to a component of X chromosome-linked miRNA cluster in the primate testis 17. Nevertheless, the expression pattern of miR-506 is contradictory in different types of malignancies, suggesting the complex role of miR-506 in different cancers. For instance, Deng et al. showed that miR-506 inhibits gastric cancer growth and metastasis by targeting YAP1 [7]. Furthermore, Arora et al. also demonstrated that miR-506 regulates epithelial mesenchymal transition in breast cancer through posttranslational control of Vimentin, Snai2 and CD151 [8]. However, Tong et al. indicated that miR-506 overexpression confers resistance to hydroxycamptothecin by inhibiting PPARα expression in colon cancer [18]. Moreover, Streicher et al. identified miRNA-506-514 as a novel oncogene in initiating melanocyte transformation and promoting melanoma growth [19]. Nevertheless, there were still inconsistent reports on the implication of miR-506 in regulating lung cancer progression. In 2011, Zhao et al. found that expression of miR-506 was reduced in 16HBE-T transformed malignant human bronchial epithelial cells compared with 16HBE normal human bronchial epithelial cells. Restoration of miR-506 in 16HBE-T cells decreased cell proliferation, cell cycle arrest and suppressed anchorage-dependent growth *in vitro* and *in vivo*. They further confirmed that miR-506 may function as a potential tumor suppressor through its targeting effect on N-Ras [9]. However, Yin et al. indicated that miR-506 was upregulated in NSCLC and functions a pro-apoptotic factor. Mechanistic investigation manifested that miR-506 directly targets NF-κB p65 to induce apoptosis of lung cancer cells [10]. To this end, we attempted to identify the potential role of miR-506 in NSCLC. The results showed that miR-506-3p expression was remarkably downregulated in NSCLC tissues and cell lines, and its expression was significantly correlated with larger tumor size, lymph node metastasis and TNM stage. In addition, we also found that miR-506-3p overexpression in NSCLC cells greatly restrained cell proliferation, migration and invasion and promoted cell apoptosis. These data collectively suggested that miR-506-3p may act as a novel biomarker for NSCLC.

It is well known that miRNA exerts its biological function by post-transcriptional regulating gene expression. Therefore, we screened target gene of miR-506-3p by using public bioinformatics algorithm. In our unpublished microarray data, we identified COTL-1 as an oncogene in NSCLC. Surprisingly, COTL1 was also identified as a potential target of miR-506-3p based on putative target sequences at position 669-676 of the COTL1 3′UTR. Dual-luciferase assay further confirmed that COTL1 is a direct target of miR-506-3p. Moreover, we found that overexpression of COTL1 restrained cell growth in cell transfected with miR-506-3p compared with that in cells transfected with miR-NC. COTL1 is an F-actin-bind protein predominantly expressed in placenta, lung and kidney [20]. In 2009, Chen et al. identified COTL1 as potential tumor antigen by immunoscreening the urinary bladder cancer cDNA library [21]. In 2011, Jeong HC et al. performed proteomics analysis to detect biomarkers between human NSCLC and normal bronchial epithelium. They found most NSCLC tissue was COTL1 positively in immunohistochemistry, western blot and RT-qPCR analysis [22]. However, the clinical significance of COTL1 in NSCLC tissue continues to be poorly understood. Moreover, whether abnormal COTL1 expression are correlated with poor prognosis in NSCLC patients is largely unknown. In the current study, we found COTL1 expression was significantly upregulated in NSCLC tissues and cell lines, with its mRNA expression negatively correlated with miR-506-3p expression. More importantly, knockdown of COTL1 mimicked the tumor-suppressive effects of miR-506-3p overexpression in A549 cells, whereas COTL1 overexpression enhanced the tumorigenic function in HCC827 cells. These combined data demonstrated that miR-506-3p exerted its tumor suppressor role in NSCLC partly by inhibiting COTL1 expression.

To elucidate the upstream signaling responsible for the reduction of miR-506-3p in NSCLC, we searched the possible transcriptional factors of miR-506 using publicly available online systems. The bioinformatics analysis showed that GATA3 has a putative binding site from −599 to −611 in the promoter region of mature miR-506. GATA3 is a zinc-binding transcriptional factor that specifies and maintains luminal epithelial cell differentiation in the mammary gland. Loss of GATA3 is involved in breast cancer pathogenesis, and a low GATA3 level is associated with higher histological grade, positive lymph nodes and poor prognosis 23. Besides, GATA3 was also downregulated in prostate, urothelial carcinoma [24, 25]. Gao et al. demonstrated that tobacco smoking might regulate the methylation of lung cancer genes, including GATA3 [26]. In addition, a few miRNA were recently identified to be directly regulated by Nrf2 to regulate tumor progression. In 2014, Chou and colleagues identified that GATA3 modulates miR-29b expression by binding to its core promoter [27]. In addition, Jiang et al. and Wang et al. demonstrated that GATA3 transcriptional actives the expression of miR-503 and miR-573 in prostate cancer [28, 29]. Nevertheless, we still have limited knowledge of aberrant expression and possible mechanisms of GATA3 in NSCLC patients. To this end, we identified that GATA3 directly binds to the promoter of mature miR-506. Ectopic expression of GATA3 promotes the miR-506-3p levels in A549 cells, whereas silencing expression of GATA3 inhibits the miR-506-3p expression in HCC827 cells. Reduced GATA3 expression was found in advanced NSCLC tissues and was positively correlated with miR-506-3p expression. Patients with lower miR-506-3p and lower GATA3 had a shorter overall survival time compared with patients with higher GATA3 and higher miR-506-3p expression. These findings indicated that reduced GATA3 levels would diminish miR-506-3p expression, and eventually enhance NSCLC progression.

LncRNA, a new class of non-coding RNA with over 200 nucleotides in length, has been receiving increase attentions, especially in cancer. Accumulating evidence indicated that lncRNA regulates gene expression at various levels, including chromatin modification, transcription and post-transcription [30]. Human UCA1 was first identified in human bladder carcinoma, and also reported in other human cancers [31–33]. UCA1 overexpression promotes cancer progression by regulating different pathways, including PI3K, Wnt and mTOR-STAT3 signaling pathway [34, 35]. Interestingly, UCA1 can also function as a ceRNA in cancer cells by interacting with miRNA. For example, UCA1 can drives breast cancer cell growth and apoptosis by inhibiting tumor suppressive miRNA-143 [36]. UCA1 promotes bladder cancer progression by targeting miR-1, miR-16 and miR-145 [37–39]. Evaluated UCA1 contributes to progression of hepatocellular carcinoma through decreasing miR-216b and activation the FGFR1/ERK signaling pathway. In addition, Bian et al. identified UCA1 promoted cell proliferation and 5-fluorouracil resistance in colorectal cancer by inhibiting miR-204-5p [40]. More recently, Nie and colleagues demonstrated that UCA1 exerts oncogenic functions in NSCLC by targeting miR-193a-3p [41]. However, whether abnormal UCA1 expression involved in miR-506 mediated NSCLC progression still unexplored. In the present study, we identified that UCA1 as an oncogenic player to bind to miR-506-3p. Abnormal UCA1 expression can modulate NSCLC cells proliferation, invasion and migration by abrogating the endogenous miR-506-3p levels. Besides, UCA1 was found to be significantly upregulated in NSCLC tissues, and UCA1 expression was negatively correlated with miR-506 levels. Patients with higher UCA1 and lower miR-506-3p had a worse overall survival time compared to those of patients with lower UCA1 and higher miR-506-3p. These combined data demonstrated that UCA1 functions as miRNA-506-5p sponge to promote NSCLC progression.

**Figure 7:**
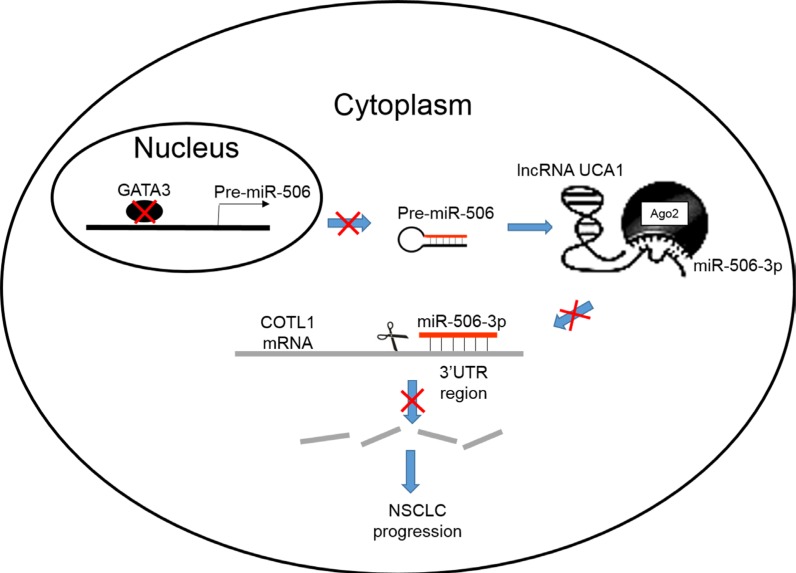
A schematic diagram deciphering the mechanism underlying GATA3 and UCA1 driven expression of miR-506-3p enhances COTL1 oncogene expression to foster NSCLC progression

In summary, we demonstrated that miR-506-3p expression was significantly downregulated in NSCLC tissues and cell lines; its expression was negatively associated with lymph node metastasis and advanced TNM stage. In addition, we also found that miR-506-3p overexpression in NSCLC cells inhibited cell proliferation, mobility, migration and invasion and promoted cell apoptosis. Furthermore, we also found that miR-506-3p exerts its function by directly targeting COTL1. Knockdown of COTL1 mimicked the tumor-suppressive effects of miR-506-3p overexpression in A549 cells, whereas COTL1 overexpression enhanced the tumorigenic function in HCC827 cells. Importantly, we also found that GATA3 transcriptionally actives miR-506-3p expression, and UCA1 is also involved in regulating COTL1 expression through competitively ‘sponging’ miRNA-506-5p. These findings indicated that genetic and epigenetic silencing of miR-506-3p enhances COTL1 oncogene expression, thus providing further insights into the mechanisms of NSCLC progression (Figure 7).

## MATERIALS AND METHODS

### Clinical specimens

Fifty-two human NSCLC and their adjacent non-cancerous tissues were collected from patients with NSCLC at First Teaching Hospital of Tianjin University of Traditional Chinese Medicine between July 2012 and April 2013. None of the patients received radiotherapy, chemotherapy, or other anticancer treatment before surgery. The histological features of all specimens were evaluated by pathologists according to the WHO criteria. All clinical-pathologic features of NSCLC patients were harvested. Informed consent was collected from each participant. All the methods in this study were in accordance with approved guidelines and all experimental protocols were approved by the Ethics Committees of First Teaching Hospital of Tianjin University of Traditional Chinese Medicine.

### Cell culture

NSCLC cell lines (A549, HCC827 and SPC-A1) and non-tumor cell line (BEAS-2B) were obtained from the American Type Culture Collection (ATCC, Manassass, VA, USA). Cells were cultured in RPMI-1640 medium (Gibco, USA) supplemented with 10% fetal bovine serum (Hyclone, USA) and 100 U/ml penicillin-streptomycin (Hyclone, USA) at 37°C under a humidified atmosphere of 5% CO2.

### Cell transfection

miR-506-3p mimics, miR-506-3p inhibitor and its negative control were purchased from GenePharma (Shanghai, China). siRNA against COTL1, GATA3 and UCA1 and scramble control were also obtained from GenePharma (Shanghai, China). Constructs for forced expression of coactosin-like protein 1 (COTL1) with or without of 3′UTR (COTL1-wt 3′UTR and COTL1-no 3′UTR) were inserted into p3′FLAG-CMV-9 expression vector. For target overexpression, the human GATA3 and UCA1 were also cloned into pcDNA3.1 vector (Invitrogen, USA). Plasmids and siRNA transfection were performed using Lipofectamine^®^ 2000 following the instructions of the manufacturer (Invitrogen, USA).

### Cell proliferation

Cell proliferation was determined by the Alarmar Blue assay following the protocol (Sigma, USA). Briefly, cells were seeded in 96-well plates and starved in medium with 1% serum overnight. After 24 h treatment, resazurin was added into culture media at a final concentration of 10%, and then cells were cultured for additional 2 h. Thereafter, cells were washed with phosphate-buffer saline (PBS) three times and then fluorescence intensity was measured using a microplate reader. The excitation and emission wavelength was 530 and 590 nm, respectively.

### Colony formation assay

For colony formation assay, 100 cells were seeded in six-well and allowed to grow in RPMI-1640 medium containing 10% FBS for 14 days. Then, cells were fixed with 4% paraformaldehyde and stained with crystal violet for 15 min at room temperature. Images were captured digitally and colonies were counted.

### Apoptosis assay

Cells after transfection were collected and washed twice with PBS. Thereafter, cells were stained with 5 μL of Annexin V-FITC and 5 μL of PI for 15 min following the instructions from the manufacturer (BD Biosciences), followed by flow cytometry analysis.

### Wound healing assays

Cell mobility was examined by a wound-healing assay. Briefly, transfected cells were cultured in six-well plates until full confluence. The wound was carefully created by scratching a confluent cell monolayer of cells using 200 μl pipette tip and washed twice with fresh medium. After 24 h treatments, the wound edges were photographed under an inverted-phase microscope and measured accordingly.

### Cell migration and invasion assay

Twenty thousand cells were seeded into the upper of the 24-well cell culture inserts with 8.0 μm probes (BD Falcon). Cell migration and invasion assay were performed with un-coated Matrigel (migration) and coated Matrigel (invasion). After 24 h, cells on the upper surface of the filters were removed with a cotton swab. For visualization, cells on lower filter surfaces were stained with 1 × DAPI solution after cold methanol fixed. Cell nuclei on the filters were visualized under a fluorescent microscope.

### RNA extraction and quantitative real-time PCR analysis (qRT-PCR)

Total RNAs from tissues and cells were isolated with Trizol according to the manufacturer's protocols (Invitrogen, USA). Total RNAs (2 μg) were then reverse transcribed into cDNA using M-MLV reverse transcriptase (Promega, USA). Gene expression was evaluated using SYBR Green qPCR master mix (Roche, USA). U6 and GAPDH were used as an internal control for miRNA and mRNA, respectively. Primer sequences were listed in Supplementary Table S1.

### Luciferase reporter assay

The complementary DNA fragment containing the 3′UTR of COTL1 was sub-cloned downstream of the luciferase gene within the pGL3-Baisc luciferase reporter vector (Promega). A549 cells were co-transfected with a reporter plasmid (100 ng) and miR-506-3p minic/miR-NC minic. Forty-eight hours after transfection, luciferase assay was determined using the Dual-Luciferase Kit (Promega). The relative firefly luciferase activities were normalized to those of Renilla luciferase.

### Western blot analysis

Tissue and cells were collected after washing with cold PBS and then lysed in RIPA lysis buffer (Solarbio) with protease inhibitor cocktail (Roche). Protein concentration was determined with a Lowry protein concentration detection kit (Solarbio). An equal amount of proteins for each sample was subjected to 8–15% sodium dodecyl sulfate-polyacrylamide gel electrophoresis (SDS-PAGE), and then transferred to nitrocellulose membranes. Antibodies were GATA3 (1:500, Proteintech), COTL1 (1: 200, Proteintech), N-Ras (1:500, Proteintech) and GAPDH (1:1000, Proteintech).

### ChIP assay

Cells were collected for ChIP by EZ-ChIP kit (Millipore, USA), as described previously [42]. Primer sequences were listed in Supplementary Table S1.

### RIP assay

A549 cells were used to perform RIP experiments using Ago2 antibody (Cell signaling) and the Magna RIPTM RNA-Binding Protein Immunoprecipitation (Millipore, USA) according to the manufacturer's instructions. After the antibody was recovered using protein A + G beads, qRT-PCR was performed to detect UCA1 and miR-506-3p in the precipitates.

### Statistical analysis

SPSS 17.0 was used for statistical analysis, and the data are presented as the means ± SD from at least three independent experiments. Difference between groups were assessed by Student's *t* test and *χ* test. Spearman correlation analysis was used to calculate the correlation between miR-506-3p and other clinical factors. The Kaplan-Meier method test was utilized for survival analysis. A *P-value* of < 0.05 determined statistical significance.

## SUPPLEMENTARY MATERIALS



## Abbreviations

miR-506-3p: microRNA-506-3p
NSCLC: non-small cell lung cancer
COTL1: coactosin-like protein 1
lncRNA: long non-coding RNA
UCA1: urothelial carcinoma-associated 1
qRT-PCR: RNA extraction and quantitative real-time PCR analysis
ChIP: Chromatin imunoprecipitation
RIP: RNA immuneprecipitation
