# Supplementary material for: Genetic and epigenetic silencing of mircoRNA-506-3p enhances COTL1 oncogene expression to foster non-small lung cancer progression
Source: Oncotarget. 2016 Nov 22;8(1):644–57. doi: 10.18632/oncotarget.13501 (PMC5352185; doi:10.18632/oncotarget.13501)
Supplement: Supplementary file 1 [file oncotarget-08-644-s001.pdf]

Genetic and epigenetic silencing of mircoRNA-506-3p enhances COTL1 oncogene expression to foster non-small lung cancer progression

Supplementary Materials

Supplementary Table S1: Primer list

qRT-PCR		
GAPDH	forward	GGAGCGAGATCCCTCCAAAAT
	reverse	GGCTGTTGTCATACTTCTCATGG
U6	forward	GTGCTCGCTTCGGCAGCACA
	reverse	GCAAATTCGTGAAGCGTTCC
COTL1	forward	AGTTGACGGAGGGAGTATTT
	reverse	CAGACGCAGGACTGAAGC
GATA3	forward	GCCCCTCATTAAGCCCAAG
	reverse	TTGTGGTGGTCTGACAGTTCG
UCA1	forward	TTTATGCTTGAGCCTTGA
	reverse	CTTGCCTGAAATACTTGC
ChIP-qPCR		
GATA3	forward	AAGAATGTGATAGCAACG
	reverse	AGCTCCAAACAATGACAG

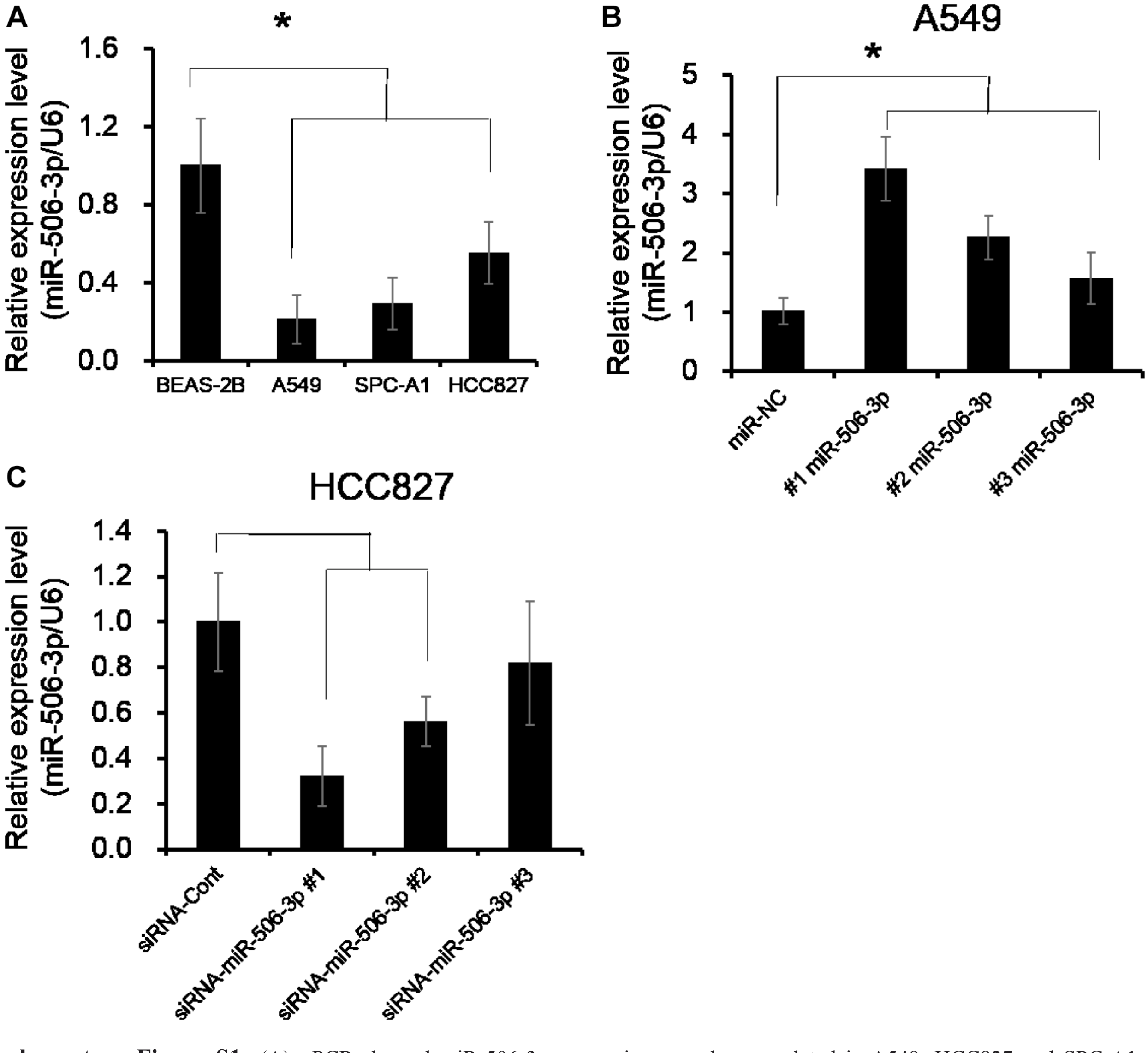

Supplementary Figure S1: (**A**) qPCR showed miR-506-3p expression was downregulated in A549, HCC827 and SPC-A1 cells compared with that of BEAS-2B cells. (**B**) qPCR showed miR-506-3p were upregulated when transfected by different miR-506-3p mimics in A549 cells. #1 miR-506-3p was selected in the present study. C. qPCR showed miR-506-3p were downregulated when transfected by different miR-506-3p siRNAs in HCC827 cells. siRNA-miR-506-3p #1 was selected in the present study. Asterisk (*) indicated $P < 0.05$.

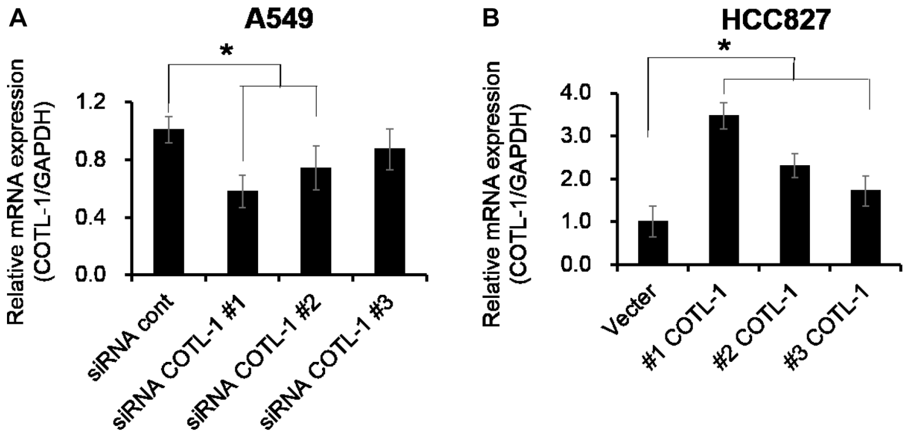

Supplementary Figure S2: (**A**) qPCR showed COTL1 was downregulated when transfected by different COTL1 siRNAs in A549 cells. siRNA COTL1 #1 was selected in the present study. (**B**) qPCR showed COTL1 was upregulated when transfected by different miR-506-3p plasmids in HCC827 cells. #1 COTL1 was selected in the present study. Asterisk (*) indicated $P < 0.05$.

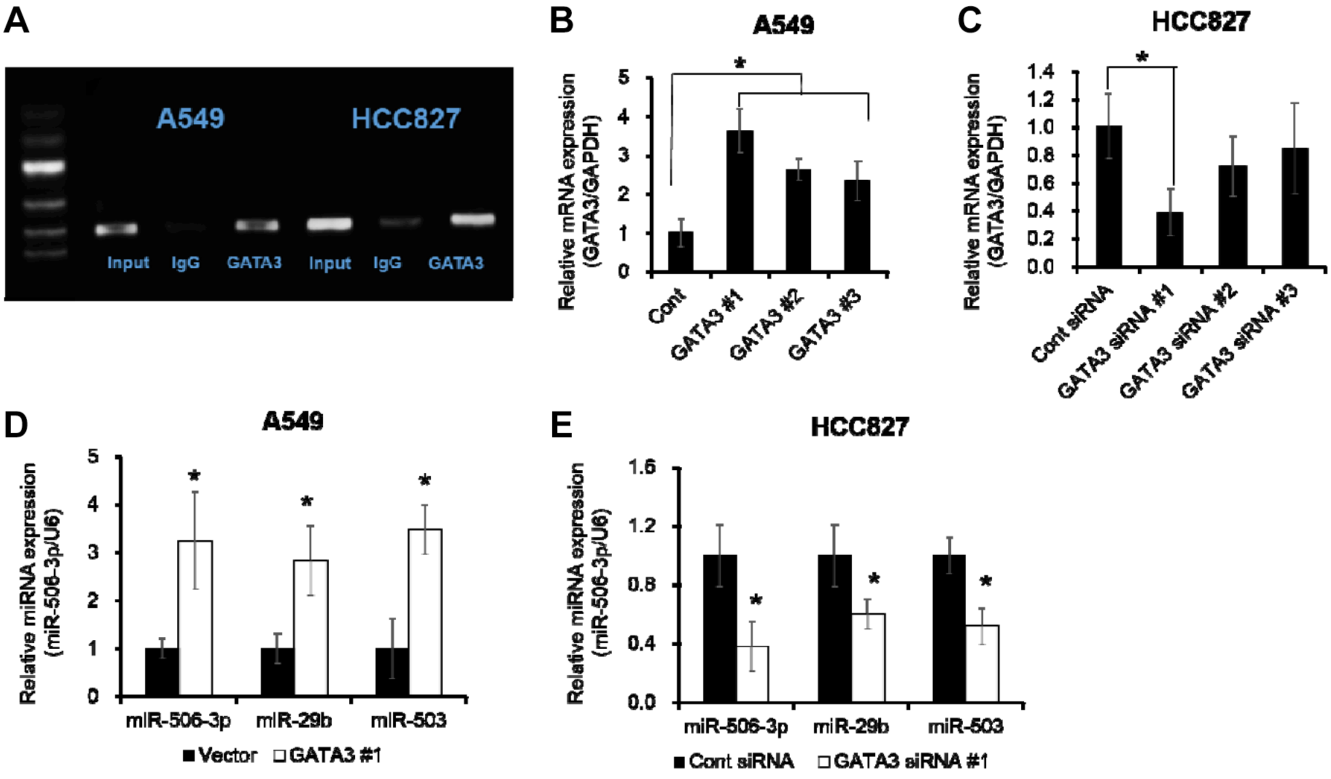

Supplementary Figure S3: (**A**) ChIP-PCR showed the levels of GATA3 at mature miR-506 promoter region in A549 cells and HCC827 cells. (**B**) qPCR showed GATA3 were upregulated when transfected by different GATA3 plasmids in A549 cells. GATA3 #1 was selected in the present study. (**C**) qPCR showed GATA3 were downregulated when transfected by different GATA3 siRNAs in HCC827 cells. GATA3 siRNA #1 was selected in the present study. (**D**) qPCR showed miR-506-3p miR-29b and miR-503 were upregulated in GATA3-transfected A549 cells. (**E**) qPCR showed miR-506-3p, miR-29b and miR-503 were downregulated when transfected by GATA3 siRNA in HCC827 cells. Asterisk (*) indicated $P < 0.05$.

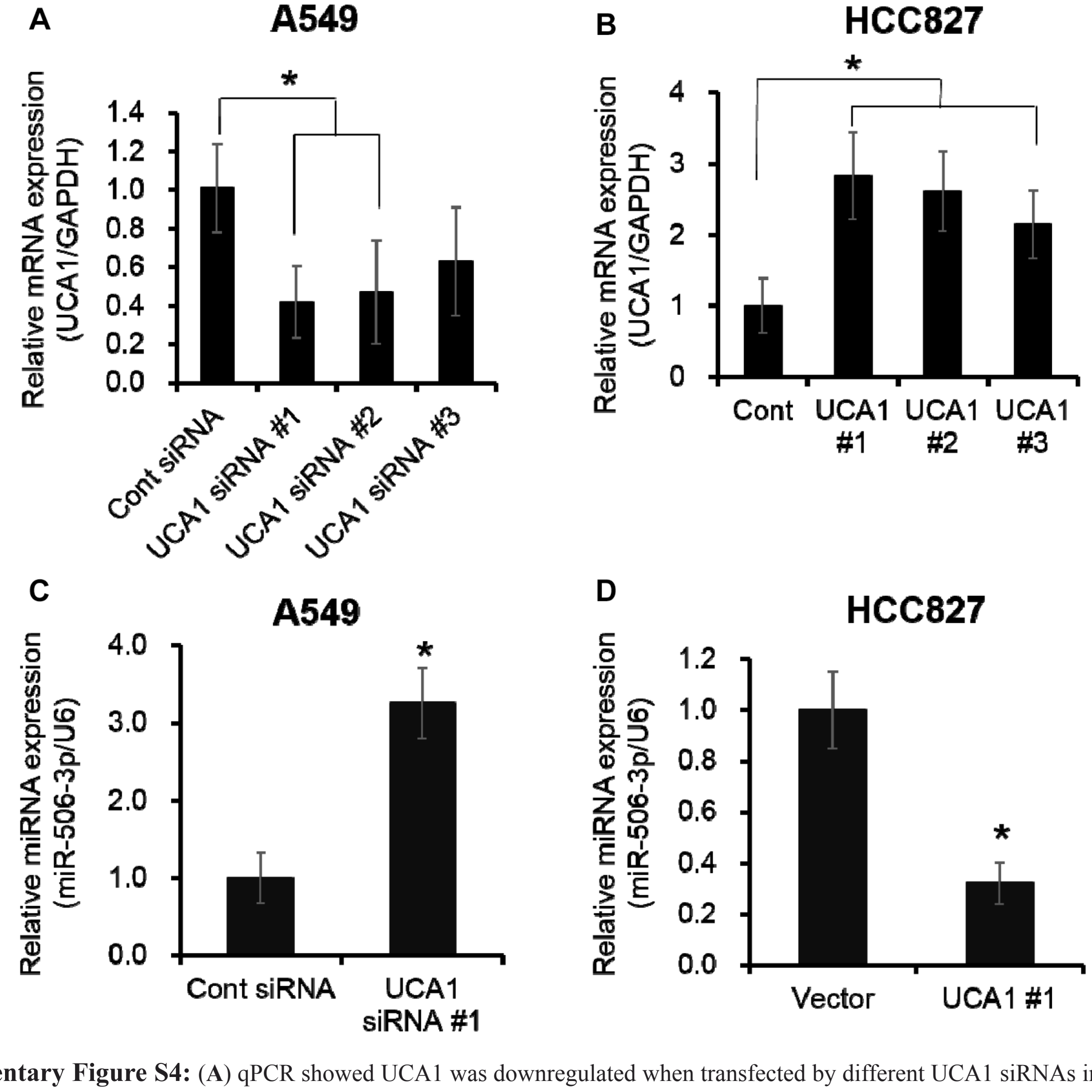

Supplementary Figure S4: (**A**) qPCR showed UCA1 was downregulated when transfected by different UCA1 siRNAs in A549 cells. siRNA UCA1 #1 was selected in the present study. (**B**) qPCR showed UCA1 were upregulated when transfected by different UCA1 plasmids in HCC827 cells. UCA1 #1 was selected in the present study. (**C**) qPCR showed miR-506-3p was upregulated when transfected by UCA1 siRNA in A549 cells. E. qPCR showed miR-506-3p was downregulated in UCA1-transfected HCC827 cells. Asterisk (*) indicated $P < 0.05$.
